# Supplementary material for: Plant-Based Diet and Glycemic Control in Type 2 Diabetes: Evidence from a Thai Health-Promoting Hospital
Source: Nutrients. 2024 Feb 23;16(5):619. doi: 10.3390/nu16050619 (PMC10934705; doi:10.3390/nu16050619)
Supplement: Supplementary file 1 [file nutrients-16-00619-s001.zip › nutrients-2850891-supplementary.pdf]

Supplemental Material: Plant-Based Diet and Glycemic Control in Type 2 Diabetes: Evidence from a Thai Health-Promoting Hospital.

Jonah B. Adokwe, Donrawee Waeyeng, Kanyamon Suwan, Kanchana Camsanit, Chanakan Kaiduong, Pawida Nuanrat, Phisit Pouyfung, Supabhorn Yimthiang, Jaruneth Petchoo, Soisungwan Satarug and Tanaporn Khamphaya

Table S1: Description of Controls and Participants with Type 2 Diabetes

Parameters	Non-diabetic controls *n*=74	Participants with Type 2 diabetes *n*=61	*p*-value
**Education, n (%)**			
Primary School	50 (67.6)	51 (83.6)	0.068
Secondary School	18 (24.3)	9 (14.8)	
College/university	6 (8.10)	1 (1.60)	
**Occupation, n (%)**			
Unemployed	12 (16.2)	11 (18.0)	0.067
Merchant	13 (17.6)	20 (32.8)	
Livestock worker	3 (4.10)	2 (3.30)	
Labourer	14 (18.9)	12 (19.7)	
Fishnet knitter	10 (13.5)	11 (18.0)	
Farmer	20 (27.0)	5 (8.20)	
**Income (THB/month)**			
<9000	46 (62.2)	40 (65.6)	0.682
≥9000	28 (37.8)	21 (34.4)	
**Exercise**	41 (55.4)	31 (50.8)	0.595

Table S2: Average Consumption Rates of Plant Foods in Five Plant Food Categories.

Parameter	Average Food Consumption (g/kg b.w./day)
**Total PFS**	
Low	4.45 ± 0.31
Medium	7.38 ± 0.22
High	10.65 ± 0.49
**Vegetables**	
Low	0.24 ± 0.04
Medium	1.17 ± 0.07
High	2.36 ± 0.13
**Fruits**	
Low	0.39 ± 0.08
Medium	1.57 ± 0.10
High	3.27 ± 0.35
**Legumes**	
Low	0.11 ± 0.03
Medium	0.39 ± 0.04
High	0.98 ± 0.13
**Nut/Seeds**	
Low	0.00 ± 0.00
Medium	0.06 ± 0.02
High	0.25 ± 0.04
**Cereals**	
Low	3.10 ± 0.24
Medium	4.21 ± 0.20
High	5.44 ± 0.26
